# *Prevotella* species as oral residents and infectious agents with potential impact on systemic conditions

**DOI:** 10.1080/20002297.2022.2079814

**Published:** 2022-05-26

**Authors:** Eija Könönen, Dareen Fteita, Ulvi K. Gursoy, Mervi Gursoy

**Affiliations:** Institute of Dentistry, University of Turku, Turku, Finland

**Keywords:** *Prevotella*, oral cavity, commensal, pathobiont, pathogen, virulence, host response, biofilm, dysbiosis, infection, systemic disease

## Abstract

Oral *Prevotella* are known as anaerobic commensals on oral mucosae and in dental plaques from early life onwards, including pigmented *P. melaninogenica, P. nigrescens,* and *P. pallens* and non-pigmented *Prevotella* species. Many *Prevotella* species contribute to oral inflammatory processes, being frequent findings in dysbiotic biofilms of periodontal diseases (*P. intermedia, P. nigrescens*), cariotic lesions (*P. denticola, Alloprevotella* (formerly *Prevotella*) *tannerae*), endodontic infections (*P. baroniae, P. oris, P. multisaccharivorax*), and other clinically relevant oral conditions. Over the years, several novel species have been recovered from the oral cavity without knowledge of their clinical relevance. Within this wide genus, virulence properties and other characteristics like biofilm formation seemingly vary in a species- and strain-dependent manner, as shown for the *P. intermedia* group organisms (*P. aurantiaca, P. intermedia, P. nigrescens*, and *P. pallens*). Oral *Prevotella* species are identified in various non-oral infections and chronic pathological conditions. Here, we have updated the knowledge of the genus *Prevotella* and the role of *Prevotella* species as residents and infectious agents of the oral cavity, as well as their detection in non-oral infections, but also gathered information on their potential link to cancers of the head and neck, and other systemic disorders.

## Introduction

Anaerobic bacteria constitute a significant part of oral microbial communities and are fundamental for oral homeostasis. The size of the core community of the mouth is estimated to be larger than those of the gut and skin [1]. Among the oral microbiota, *Bacteroidetes* is one of the major phylae and *Prevotella* its largest genus [2]. This genus, described by Shah and Collins in 1990 [3], consists of pigmented or non-pigmented, Gram-negative, strictly anaerobic, short rod-shaped bacteria. Besides 12 former *Bacteroides* reclassified as *Prevotella* species [3], a high number of *Prevotella* species of oral origin from humans have been described. A recent whole-genome analysis of *Prevotella* genomes available in public repositories demonstrated variable genome lengths (2.37–4.26 Mb) and G + C contents (36.4–56.1%) between the 25 human *Prevotella* species included in the analysis [4], indicating a wide diversity within the genus. In addition, a closely related novel genus *Alloprevotella* consists of two oral species, *A. tannerae* (formerly *Prevotella tannerae*) and *A. rava* (formerly known as *Prevotella* oral taxon 302) [5]. Knowledge of such a variety of species and their impact on the oral microbiota and human well-being is of concern.

Many *Prevotella* species are common residents on various surfaces of the mouth, often with a commensal relation to their host. One example is *P. melaninogenica*, a ubiquitous species in the oral cavity, where it colonizes mucosal surfaces of infants from the early months of life onwards [6,7]. Other early-colonizing oral *Prevotella* species are *P. nigrescens* and *P. pallens* [8]. While the latter species of the *P. intermedia* group is without any specific connection to oral diseases, *P. nigrescens* and *P. intermedia*, in particular, show moderate pathogenicity, being present in periodontitis-associated subgingival complexes [9]. A concept of dysbiotic biofilms, where commensals can behave as opportunistic pathogens in susceptible hosts, is useful to describe oral infection-driven inflammatory diseases like periodontitis [10]. Cooperative or competitive interactions between bacteria regulate the composition of biofilms and their properties. Since *Prevotella* is a highly diverse genus [4], some of its species could behave as true commensals but some as potential pathobionts, which trigger inflammatory mediators with harmful consequences not only in the mouth but also at extra-oral sites. For instance, proinflammatory interleukin-17 produced in response to *P. nigrescens* could aggravate the injury in arthritis-affected joints [11].

**Figure 1.**
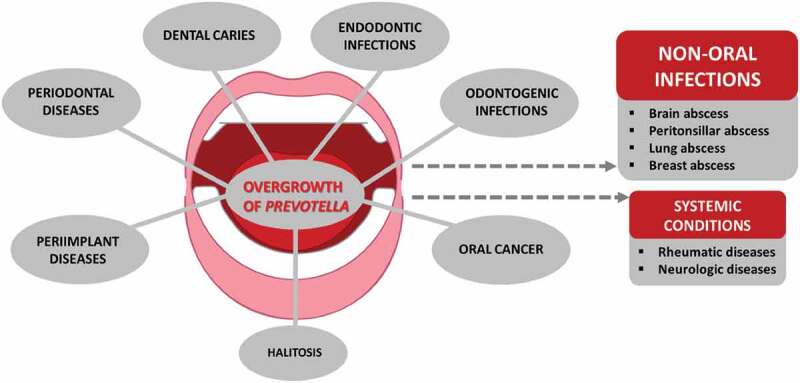
Oral *Prevotella* species detected in clinical specimens from oral and non-oral diseases/infections.

The hematogenous spread of oral bacteria, including *Prevotella*, can occur after dental procedures [12]. A novel perspective is the blood microbiome, where oral bacteria seem to have a significant impact [13], which raises a question on their potential involvement in systemic inflammatory conditions. Due to pathological processes present in the mouth, such as periodontitis, caries, and endodontic infections among others (Figure 1), many inflammatory agents produced by the host can migrate via the blood circulation to extra-oral sites [14]. Indeed, translocated oral, vaginal, and intestinal *Prevotella* species have harmful consequences in a huge variety of clinically relevant infections at the head and neck area and the gastrointestinal, lower respiratory, and urogenital tracts, skin and soft tissues, central nervous system, and blood [15]. Moreover, there is evidence that *Prevotella* may have a role in malignancies as well as in non-infectious chronic conditions like rheumatic diseases and neurodegenerative disorders.

Many *Prevotella* organisms of oral origin seem to be important players in the gastrointestinal and respiratory tracts during health and disease [16]. *Prevotella* constitutes a significant part of oral microbial communities; however, species-level data are still scarce due to a considerable expansion within the genus during the past two decades. In this narrative review, we focus on the presence and properties of individual species in the oral cavity, by gathering the knowledge of various *Prevotella* species as oral residents and disease-associated agents, as well as their potential impact on some systemic conditions.

## *Prevotella* species in the developing anaerobic microbiota and as commensals of the mouth

While the facultative genera *Streptococcus, Gemella*, and *Granulicatella* predominate in saliva of 3–4-day-old neonates, the genus *Prevotella* is still rare and its relative abundance low [17]. However, the anaerobic organisms of the genera *Veillonella* and *Prevotella* start their establishment in the mouth within 1–2 months of life, as shown in culture-based studies from the 90ʹs. Thorough anaerobic techniques and longitudinal study designs revealed the time of colonization and succession of anaerobes in the oral cavity, with *P. melaninogenica* as a common anaerobic finding on oral mucosae and in saliva of predentate infants [6,7]. For pioneer commensals, the clonal diversity seems to be typical, and up to seven simultaneous clones have been shown for early-colonizing anaerobes, *P. melaninogenica* and *Fusobacterium nucleatum* [6,18]. This wide antigenic variety and frequent turnover of strains would allow escape from the host immune response; thus, these species can persistently colonize the habitat and elicit natural immunity. In particular, the key role of *F. nucleatum* in successive colonization of other species in oral biofilms is of interest. Besides *P. melaninogenica*, the frequency of oral pigmented *Prevotella* organisms, including *P. denticola, P. loescheii, P. nigrescens*, and *P. pallens*, and non-pigmented *Prevotella* species remarkably increased during the first years of life [6,19]. The composition of parents’ oral microbiota with potential pathogens and frequent contacts reflect the developing microbiota of their children [8,20], which may affect their oral health later in life. At the clonal level, knowledge of intra-familial transmission of oral *Prevotella* between mother-child pairs or between spouses includes *P. melaninogenica, P. pallens, P. nigrescens*, and *P. intermedia* [19,21,22]. Within the *P. intermedia* group, recoveries of similar clones of *P. nigrescens* and *P. pallens* from maternal saliva and oral samples of their young children were mainly from periodontally healthy mothers [19]. A recent review [23] underlined the necessity of large-scale, detailed longitudinal studies to reveal the spatial diversity and acquisition patterns of the early oral microbiota for understanding conformities of a symbiotic host–microbiota relationship. Crielaard and co-workers [24] explored temporal changes in the microbiome that occur due to the natural development of the dentition along with age by analyzing the salivas of children with the deciduous, early mixed, late mixed, and permanent dentition. The genus *Prevotella* was among the three predominant taxa in saliva, and its relative abundance was steadily increased due to this age-driven developmental maturation [24]; however, no species-level data were available. In 240 Kuwaiti children, stratified into five age groups (<6, 6–9, 10–12, 13–15, and 16–18 years of age), the salivary carriage rates of *P. intermedia* and *P. nigrescens* were clearly distinct [25]. While the rates of *P. intermedia* remained low, varying from 2.5% in the youngest group to 6.7%, 15%, 10%, and 8.3% in the oldest group, the corresponding rates for *P. nigrescens* were much higher, i.e. 15%, 31.7%, 62.5%, 50%, and 46.7%. A similar trend was reported from saliva and subgingival samples collected from 119 healthy children aged 2–15 years in Japan [26]. A recent study compared the bacterial composition of subgingival plaque samples from the offspring of two parent groups, including 18 parents with generalized severe (grade C) periodontitis and 18 parents without periodontal disease [20]. In the core microbiome of their children aged 6–12 years, *P. maculosa, P. melaninogenica, P. oris*, and *P. oulorum* belonged to the core microbiome, determined as being present in at least 80% of the children, irrespective of the parent group. Instead, in the core microbiome of children of diseased parents, a much wider selection of *Prevotella* taxa was found, including *P. histicola, P. nanceiensis, P. nigrescens, P. pleuritidis, P. saccharolytica, P. scopos, P. veroralis*, and *A. tannerae*. Furthermore, *Prevotella* abundance differed between children of parents with and without periodontitis. The most abundant *Prevotella* species in children of periodontitis parents were *P. shahii* and *P. scopos*, with over 6- and 4-fold difference, respectively, compared to children of periodontally healthy parents. In addition, *P. denticola, P. maculosa*, and *P. pleuritidis* had over 3-fold difference, and *P. oris, P. oulorum, P. saccharolytica, P. salivae*, and *P. veroralis* over 2-fold difference [20].

In healthy adults, the detection rates of the genus *Prevotella* are high in saliva and dental plaque [17,27]. In saliva, the richness of *Prevotella* diversity was especially high [26]. A study looking for the intraoral distribution of bacterial species in 225 systemically healthy individuals showed *P. melaninogenica* in high proportions in saliva and at the dorsum and lateral sites of the tongue [28]. In the oropharynx of young adults, tonsillar crypts are colonized by a variety of *Prevotella* species, among those *P. buccae, P. dentalis, P. denticola, P. fusca, P. micans, P. oralis, P. oris, P. pallens, P. salivae*, and *P. veroralis*, typical oral species, but also *P. nanceiensis* and *P. pleuritidis*, less known residents of the oral cavity or oropharynx [29]. In the literature, the species-level data on *Prevotella* organisms have long been limited to *P. intermedia* and/or *P. nigrescens* due to their clinical relevance in oral pathologies, but also ignorance of commensals. However, this attitude is no longer valid, and knowledge of their prevalence and abundance is rapidly increasing.

In sequencing studies with limited numbers of participants, representing various age and ethnical backgrounds, *P. melaninogenica* appears among the core oral bacteriome in individuals with healthy oral tissues [30,31]. Less abundant core species included *P. denticola, P. oris*, and *A. tannerae*, while *P. multiformis* was recognized at low prevalence but relatively high abundance [31]. However, also some differences have been reported; a comparison of bacterial communities in saliva of a total of 2,815 individuals showed significantly higher relative abundances of the genus *Prevotella* and *P. melaninogenica*, in particular, in orally healthy Japanese than South Korean study participants [32]. A recent study compared the tongue microbiome of 10 urban and 10 rural women, aged 20–45 years, in Indonesia [33]. In the urban group, *Prevotella* and *Leptotrichia* were among the most abundant genera, and *P. melaninogenica, P. histicola, P. pallens*, and *P. salivae* were the most discriminative *Prevotella* species between the urban and rural groups, probably connected to different environmental and lifestyle factors. When comparing bacterial communities of the buccal mucosae of 20 young healthy individuals who smoked 15–24 cigarettes daily to those of 20 non-smokers, increased abundance of several species, including *P. salivae, P. melaninogenica* and *P. histicola*, was observed in smokers [34]. In studies dealing with Finnish periodontitis-free individuals, the most frequent pigmented *Prevotella* species were *P. melaninogenica, P. intermedia/P. nigrescens* and *P. loescheii* [35], whereas *P. intermedia* (*sensu stricto*) has been a rare finding [19,22,36]. Interestingly, *P. jejuni*, which is a pigment-producing species closely related to *P. melaninogenica*, but not originally accounted for as an oral species, proves to be present in oral specimens [33,37,38]. Recoveries from a general population-based study, looking for PCR-based carriage rates of periodontal pathogens in saliva of 1,294 adults living in southern Finland, only 13.9% were positive for *P. intermedia* [39], which was more frequent in women than in men and in smokers than in non-smokers. Similarly, a low detection rate of 10% for *P. intermedia* was reported from subgingival plaques of 504 Australian adults, mainly composed of university staff [40]. With regard to other *Prevotella* species, such information based on large study populations is limited. In the salivary microbiome of more than 2,300 inhabitants aged ≥40 years of the town of Hisayama, a demographically representative of Japan, *P. melaninogenica* was recovered as a core bacterial species, and other common *Prevotella* species were *P. denticola, P. histicola* and *P. salivae* [41]. Another study based on the same study material focused on the bacterial composition on the tongue dorsum in 506 community-dwelling elderly, accounting for half of the age group of 70–80 years living in the town [42]. *P. melaninogenica* was the second most predominant finding, but also *P. histicola* and *P. pallens* were among the predominant findings on the tongue dorsum.

## Properties of oral *Prevotella* organisms

### Species-level differences within the *P. intermedia* group

Here, this specific *Prevotella* group was selected as an example to demonstrate the wide heterogeneity of properties of biological and medical relevance even within closely related species. With this comparison, we emphasize the importance of obtaining species-level information for a better understanding of their potential roles as commensals, pathobionts, or ‘true’ pathogens.

The *P. intermedia* group is composed of four phylogenetically close species, i.e. *P. intermedia, P. nigrescens, P. pallens*, and *P. aurantiaca*. Their shared biochemical characteristics are the ability to decompose tryptophane into indole, moderate saccharolytic activities, and production of pigment [43–45]. All four species are residents of the oral cavity, which represents their predominant site of isolation. When Shah and Gharbia [43] used DNA–DNA hybridization to propose the separation of the phenotypically identical *P. intermedia* and *P. nigrescens* into two genetically distinct species, the division was not only based on their different homology groups but also on their dissimilar virulence properties. While *P. intermedia* was associated with periodontal infections, *P. nigrescens* was found at both healthy and diseased periodontal sites and as a core species mediating fluctuations in the subgingival microbiota [22,43,46]. *P. pallens* recoveries come mainly from healthy children and periodontitis-free women [19] and, in addition, it seems to be a resident species on healthy esophageal mucosae [47]. The newest member of the *P. intermedia* group, *P. aurantiaca*, was isolated from the periodontal pocket of a patient with periodontitis [45]. However, the literature is scarce regarding its involvement in oral or systemic infections.

In relation to periodontal health, the physiological and hormonal changes during pregnancy exhibit a direct impact on periodontal tissues [48]. *P. intermedia* and *P. nigrescens* can take up and utilize sex steroid hormones (estrogen and progesterone) as substitutes for vitamin K, the essential growth factor, in temperature- and concentration-dependent manners [49]. The impact of estradiol on the growth characteristics, proteolytic enzyme activities, and biofilm-related pathogenicity within the *P. intermedia* group organisms has been examined *in vitro*, partially simulating the clinical conditions of pregnancy-associated gingivitis [50,51]. *P. nigrescens, P. pallens*, and *P. aurantiaca* increased their numbers in the planktonic phase with elevated estradiol levels, and *P. intermedia, P. nigrescens*, and *P. pallens* exhibited higher protein concentrations in the presence of estradiol, presenting the ability to form biofilms. In addition, *P. intermedia* enhanced polysaccharide production and coaggregation capabilities with *F. nucleatum*, indicating their potential inter-species differences in the pathogenesis of pregnancy-associated gingivitis [50]. Estradiol affected the virulence of *P. intermedia, P. nigrescens, P. pallens*, and *P. aurantiaca*, as seen in their bacterial dipeptidyl peptidase IV (DPPIV) enzyme activity in static biofilms, in a strain- and dose-dependent manner [51,52]. Variations in the distribution of virulence-associated genes among *Prevotella* species seem to regulate their intra-oral colonization and relation to disease pathogenesis; for example, beta-lactam resistance genes significantly altered the frequency and distribution of certain strains of *P. intermedia, P. nigrescens*, and *A. tannerae* at different sites in the oral cavity [53].

### Virulence factors

Properties of *Prevotella* organisms vary due to the wide diversity of this anaerobic genus. As having a Gram-negative cell structure, lipopolysaccharide (LPS) is a potential virulence factor of *Prevotella* species. For instance, LPS of *P. intermedia* stimulates the expression of IL-8 in human gingival fibroblasts, invades the human oral epithelial cells (KB cells), impairs phagocytic and chemotactic activities in human dental stem cells, and stimulates alveolar bone resorption [54–56]. *P. intermedia* and *P. nigrescens* produce mannose polysaccharide, which allows these species to participate in chronic inflammatory processes, including the modification of human leukocyte phagocytosis and invasion of host defense barriers [57,58].

Other virulence factors are connected to fimbriae, outer membrane vesicles, protein secretion systems (enzymes and toxins), resistance to oxidative stress, and pH modification [59–61]. Indeed, *P. intermedia* carries a Type IX secretion system, allowing it to release its proteases, hemolysins, and other virulence factors on outer membrane proteins, thus dysregulating host cell responses [62]. As regards *P. intermedia* and *P. nigrescens*, the pathways of energy metabolism, carbohydrate and lipid metabolism, and amino acid metabolism differ between disease- and health-related samples [61]. Regarding surface structures, various types of *P. intermedia* fimbriae mediate their adherence to host erythrocytes to facilitate their agglutination and create a fimbriae-associated hemagglutinin activity essential for *P. intermedia* colonization *in vivo* [63]. Host cellular adhesion is an essential part of bacterial virulence; *P. intermedia* and *P. nigrescens* demonstrated representative adhesion capabilities to invade epithelial cells, with special tendency to bind the epithelial cell lamellipodia [64].

The catalytic action of proteolytic enzymes (cysteine and serine proteinases) produced by oral *Prevotella* species is considered a significant virulence factor, contributing to periodontal tissue destruction through the cleavage of cellular peptides, which leads to the degradation of collagenous matrix in the periodontium [65–67]. *Prevotella* strains isolated from oral infectious lesions, e.g. purulent periodontal pockets and peri-implantitis sites, demonstrate higher proteolytic enzyme activities than resident *Prevotella* of healthy sites [66,68]. As part of host-bacterial interactions, the degradation of immunoglobulins by total proteases of *P. intermedia* and *P. nigrescens* is strongly linked to the causality of periodontal disease originated from polymicrobial infections [69].

DPPIV is a serine protease of bacterial and host origin. Bacterial DPPIV is an endopeptidase that hydrolyzes the penultimate proline or alanine dipeptides from the N-terminal polypeptide chains, while the host CD26 is a transmembrane glycoprotein encoded by the *DPPIV* gene and is expressed on the surface of different cell types, including human gingival keratinocytes and fibroblasts [70–72]. The CD26/DPPIV plays a key role in many physiological and pathological processes, such as glucose metabolism and chemokine regulation, but also acts as a tumor marker for various types of cancers [73–75], and has a strong association with the severity of disease and comorbidity in patients with COVID-19 [76,77]. In bacteremia induced by periodontitis-associated species (*Porphyromonas gingivalis, Tannerella forsythia*, and *P. intermedia*), DPPIV shows a significant capability of modulating blood glucose levels, potentially deteriorating the health condition of diabetes, and is considered a powerful biomarker for the existence of periodontopathogens in subgingival biofilms [78]. Via an indirect pathway, the expression of virulence by *P. melaninogenica* is significantly regulated by the proteases and cell surface proteins secreted by *P. gingivalis* and *T. forsythia* as virulence factors [79].

Hemagglutinating bacteria use certain agglutinins that enable red blood cells to be clumped by serum antibodies, while the hemolytic activity of bacteria is their ability to produce hemolysins for the breakdown of red blood cells and release hemoglobin [80]. Both properties are considered virulence characteristics of bacteria with fimbriae. *P. intermedia* and *P. nigrescens* have high hemagglutinating and hemolytic activities that crucially benefit their growth and persistence, as well as to synergistically support the virulence of other pigmented species, e.g. providing haem to *P. gingivalis* in subgingival plaque through a mutualistic proteolytic conjunctive action by *P. intermedia* [81–83]. As a metabolic mechanism, oral *Prevotella* species require haem as an indispensable nutrient for their persistence and to express virulence [84]. *P. intermedia* contributes to haem acquisition by another proteolytic enzyme, interpain A or albuminase, to degrade hemoglobin in inflamed periodontal pockets under special conditions of low redox potential and high pH [83,85]. Unlike *P. intermedia*, the lactoferrin-binding protein identified in *P. nigrescens* from a periodontal pocket is seemingly an active player in its iron acquisition mechanism [86]. As part of the essential iron-scavenging utilization, the iron-saturated human transferrin-binding capacity of *P. nigrescens* allows it to survive and proliferate in deep periodontal pockets [87]. Although considered a low magnitude of virulence, *P. melaninogenica* is another example of *Prevotella* species exhibiting a hemagglutinating activity and agglutinating erythrocytes [88].

### *Prevotella* in oral (dysbiotic) biofilms

In comparison with the planktonic phase of growth, bacteria living in oral biofilms are shielded against the disruptive mechanical and chemical forces (e.g. tooth brushing, salivary flow, and antimicrobials) and are protected from phagocytic immune cells and the complement system to recognize them [89–92]. Thus, biofilm formation is among the significant virulence aspects of oral *Prevotella* species to establish a tolerable environment, optimal for their growth and proliferation [91]. Various *Prevotella* species, including *P. intermedia, P. nigrescens*, and *P. denticola*, can take part in both sugar and protein metabolisms, allowing them to survive in both health- and disease-associated conditions, and in supra- and subgingival areas [59,93]. *Prevotella* species usually colonize the dental biofilm in micro-colonies, being visible on the top layers of the biofilm [94]. Yet, there are variations in biofilm formation capabilities of *Prevotella* species in *in vitro* conditions; *P. loescheii, P. oris*, and *P. nigrescens* demonstrate better biofilm forming abilities than *P. intermedia, P. melaninogenica, P. pallens*, and *P. oulorum* [95]. A dysbiotic shift in biofilms occurs with a change in the environment, for example, with an increase in dietary sugars or in inflammatory proteins, leading to common destructive diseases, caries, and periodontitis. An inflammation-induced protein-rich environment allows the proliferation of amino acid-degrading *Prevotella* in subgingival biofilms [96]. Systemic diseases connected to a chronic inflammatory state, such as rheumatoid arthritis, induce *Prevotella* to enrich in the oral microbiota as well [97]. Indeed, enriched *Prevotella* can neutralize the acidic stress by fermenting glutamate and aspartate into ammonia, allowing acid-sensitive *P. gingivalis* and *Treponema denticola* to become more dominant in subgingival biofilms [98]. In addition to these indirect synergistic effects, cell-to-cell contacts with *Prevotella* stimulate biofilm formation: *P. intermedia* and *F. nucleatum* [99] and *P. oris* or *P. nigrescens* and *P. gingivalis* [100,101]. Not only whole cells but also metabolites of *Prevotella* can modify cellular response against bacteria. Short-chain fatty acids of *P. loescheii* inhibit murine T- and B-cell proliferation and cytokine production by splenic T cells [102]. In particular, volatile propionic, butyric, and isovaleric acids are able to induce DNA fragmentation and apoptosis in B cells [103]. Soluble sonic extracts of *P. loescheii* induce a dose-dependent inhibition of human peripheral blood lymphocyte and leukemic cell line (BALL-1) proliferations [104]. These findings indicate that *Prevotella* organisms within biofilms can regulate host residence and immune cell response, without being in need of a direct contact with the host. Although *Prevotella* species are considered to be aerotolerant [105], coaggregation with *F. nucleatum* seems to be the major determinant for *Prevotella* to survive in aerated environments [90]. Taken together, *Prevotella* species not only contribute to the biofilm’s structural maintenance but also benefit from the multispecies character of oral biofilms.

## Host cell response to oral *Prevotella*

Amongst the vast number of bacteria–host cell interaction studies, only few studies include *Prevotella* as a test organism and/or demonstrate cellular response against *Prevotella* species other than *P. intermedia*. This species is common in periodontitis-affected gingival tissues, indicating its direct interactions with periodontal resident and immune cells [106]. *P. intermedia* is a strong inducer of epithelial anti-microbial peptides [human beta-defensins (hBD-1, −2, −3)] and IL-8, especially when incubated with human gingival keratinocytes with a multiplicity of infection (MOI) of 1,000 [107]. Such an effect was not observed when *P. intermedia* was incubated with a MOI of 200, meaning that the cellular response against *P. intermedia* requires colonization and proliferation of *P. intermedia*. Not only *P. intermedia* but also its glycoproteins, but not its LPS, can activate IL-8, granulocyte colony-stimulating factor, and granulocyte-macrophage colony-stimulating factor protein expressions of gingival keratinocytes [108]. Thus, it is possible that oral *Prevotella* species do not only aggravate the disease extension and progression but also protect the healthy homeostasis in a species-dependent manner.

Cellular response against *Prevotella* is not limited to gingival keratinocytes. Indeed, *P. intermedia* activates COX-2-dependent PGE_2_ expression in periodontal ligament fibroblasts in a dose- and time-dependent manner [109]. *P. nigrescens* LPS can activate PGE_2_ expression together with transforming growth factor production in bone marrow mononuclear cells and induce osteoclastogenesis [110]. A similar dose-dependent cellular response was observed when mouse odontoblast-like cells and macrophages were incubated with *P. intermedia* LPS, which ended up elevating vascular endothelial growth factor protein expressions [111]. In fact, mouse studies produced evidence that *P. nigrescens* can induce Th17 response via toll-like receptor-2 and IL-1 activation [112]. Human dental pulp cells also react against *P. intermedia* LPS by producing IL-10 receptor [113]. *P. nigrescens* LPS increases cell surface fibronectin expression of gingival fibroblasts in inflamed gingiva [114]. Yet, the clinical significance of elevated cell surface fibronectin in periodontal disease pathogenesis is not well understood. Finally, *P. intermedia* can stimulate CD4(+) T cell subsets to produce cytokines interferon-γ and IL-2 [115]. Since these cytokines are expressed by Th1 lymphocytes, it is possible that *P. intermedia* regulates T lymphocyte differentiation.

As seen here, the main gap in the literature is within the interactions between human cellular response and oral health-associated *Prevotella* commensals. Further studies are needed to demonstrate how different *Prevotella* species contribute to a healthy oral equilibrium.

## *Prevotella* involvement in oral conditions of clinical relevance

Besides being a core species in various locations of the oral cavity, many members of the genus *Prevotella* are common findings in clinical specimens collected from oral infections (Figure 1). In early studies, *Bacteroides melaninogenicus* subspecies *intermedius* was connected to plaque-induced gingival inflammation and periodontitis [116,117] as well as to specific types of gingival diseases, such as pregnancy gingivitis and necrotizing ulcerative gingivitis [36,118]. After the description of the genus *Prevotella* [3] and separation between *P. intermedia* and *P. nigrescens* [43], it was soon observed that these species differ as regards to their site specificity, surface properties, enzyme activities, and pathogenicity [46,119]. While the majority of *P. intermedia* originated from deepened periodontal pockets, only a few strains came from endodontic infections, and *P. nigrescens* was common at healthy gingival sites [46,119]. In subgingival biofilms, however, both species belong to the orange complex with a moderate association with periodontal disease [9].

The recoveries of specific *Prevotella* species from a variety of oral diseases have been gathered in Table 1.

**Table 1.** Detection of specific *Prevotella* species (validly published) of oral origin in oral and non-oral diseases/conditions

*Prevotella* sp. of oral origin	Primary isolation site as described	Description reference	Recoveries from oral diseases/infections	Recoveries from non-oral diseases/infections and potential role in systemic conditions*	References
*P. aurantiaca*	Periodontal pocket	[45]	ND	Immunoglobulin A nephropathy	[232]
*P. baroniae*	Endodontic lesion, dentoalveolar abscesses, pericoronitis lesion, subgingival plaque	[233]	Dentin caries Endodontic infections Periodontitis Endodontic abscesses Severe odontogenic abscesses	Brain abscesses	[127,138,148–150,154,156,162,190,191]
*P. buccae*	Periodontal pocket	[3]	Dentin caries Endodontic infections Radicular cyst Periodontitis Periodontal abscesses Severe odontogenic abscesses	Head and neck infections Descending necrotizing mediastinitis Pleural empyemas Breast abscesses Infected breast cyst	[123,138,150,151,157,160,161,192,195–197,234–237]
*P. buccalis*	Oral cavity	[3]	Endodontic infections Periodontitis	Head and neck infections	[235,236,238]
*P. dentalis* (formerly *Mitsuokella dentalis*)	Dental root canal	[239]	Dentin caries Endodontic infections Periodontitis Periodontal abscesses	Head and neck infections Early-onset rheumatoid arthritis*	[136,138,150,157,205,235]
*P. denticola*	Oral cavity	[3]	Early childhood caries Dentin caries Root caries Endodontic infections Periodontitis Periodontal abscesses Severe odontogenic abscesses Peri-implant mucositis Peri-implantitis	Head and neck infections Brain abscesses Diabetic foot osteomyelitis Rheumatoid arthritis*	[122–125,133,134,136,139–142,145–147,157,161,190,191,195,200,217,234,235,240]
*P. enoeca*	Gingival crevice (healthy gingiva), periodontal pocket	[241]	Periodontitis Peri-implant mucositis	Anaerobic meningitis	[127,131,242]
*P. fusca*	Periodontal pocket	[243]	Periodontal abscesses Peri-implantitis	Diabetic foot osteomyelitis	[136,157,200]
*P. heparinolytica*	Periodontal pocket	[3]	Periodontal abscesses	Brain abscesses	[158,190,191]
*P. histicola*	OSCC, oral mucosae	[244]	Early childhood caries Dentin caries Periodontitis Periodontal abscesses	Rheumatoid arthritis*	[42,125,138,140,157,217]
*P. intermedia*	Oral cavity (incl. periodontal pocket)	[43]	Dentin caries Endodontic infections Radicular cyst Endodontic abscesses Periodontitis Periodontal abscesses Severe odontogenic abscesses Peri-implantitis Alveolar osteitis (“dry socket”) Primary Sjögren sdr and dry mouth Oral cavity leukoplakia OSCC	Laryngeal cancer Brain abscesses Head and neck infections Hidradenitis suppurativa (acne inversa) Diabetic foot osteomyelitis Early-onset rheumatoid arthritis* Alzheimer´s disease*	[123,124,126,128,133,134,138,147,150,151,153,154,156–158,161–163,167,177,178,181,187,190,191,195,198,200,205,220,221,234,235,240,245]
*P. loescheii*	Gingival crevice	[3]	Periodontitis Peri-implant mucositis Peri-implantitis OSCC	Head and neck infections Septic arthritis Infection of hip arthroplasty	[124,161,180,235,236,240,246,247]
*P. maculosa*	Pericoronitis, peri-implantitis, subgingival plaque	[248]	Dentin caries Periodontitis Peri-implantitis Alveolar osteitis (“dry socket”)	Rheumatoid arthritis*	[124,138,144,163,217,240]
*P. marshii*	Endodontic lesion, supragingival plaque subgingival plaque	[233]	Dentin caries	ND	[138]
*P. melaninogenica*	Oral cavity, throat, tonsils	[3]	Early childhood caries Dentin caries Endodontic infections Endodontic abscesses Pregnancy gingivitis Periodontitis Periodontal abscesses Severe odontogenic abscesses Peri-implantitis Halitosis Alveolar osteitis (“dry socket”) Oral lichen planus Sjögren sdr and focal sialoadenitis OSCC	Tonsillar SCC Oropharyngeal and hypopharyngeal SCC Head and neck infections Hidradenitis suppurativa (acne inversa) Rheumatoid arthritis* Alzheimer´s disease*	[121,124,125,128,134,140,143,148,157,161,163,164,168,171,183,185,186,195,198,217,218,221,234,235,249]
*P. micans*	Necrotic pulp, periodontal pocket, gingival crevice	[250]	Peri-implant mucositis Peri-implantitis	ND	[240]
*P. multiformis*	Oral cavity	[251]	Endodontic-periodontal lesions Periodontitis Periodontal abscesses Peri-implantitis Alveolar osteitis (“dry socket”)	ND	[123,124,136,157,163,252]
*P. multisaccharivorax*	Periodontal pocket	[253]	Dentin caries Root caries Endodontic infections Endodontic abscesses Peri-implant mucositis OSCC	ND	[131,138,145,146,149,154–156,182]
*P. nigrescens*	Oral cavity (incl. periodontal pocket)	[43]	Dentin caries Endodontic infections Endodontic abscesses Pregnancy gingivitis Periodontitis Periodontal abscesses Peri-implantitis Alveolar osteitis (“dry socket”) OSCC	Oropharyngeal and hypopharyngeal SCC Laryngeal cancer Brain abscesses Hidradenitis suppurativa (acne inversa) Rheumatoid arthritis* Systemic lupus erythematosus* Alzheimer´s disease*	[36,53,121,124,126,128,132,134,135,138,144,147,148,150,153,154,156,157,163,183,186,187,190,191,198,206,217,221]
*P. oralis*	Gingival crevice	[3]	Dentin caries Endodontic infections Endodontic abscesses Periodontitis Dry mouth	Head and neck infections	[123,124,138,150,154,161,167,195,235,236,238]
*P. oris*	Periodontal pocket	[3]	Dentin caries Endodontic infections Pregnancy gingivitis Periodontitis Periodontal abscesses Severe odontogenic abscesses Peri-implantitis OSCC	Laryngeal cancer Head and neck infections Brain abscesses Pleural empyemas Systemic lupus erythematosus*	[121,123,124,133–135,141,144,148,150,157,160–162,177,187,190–192,206,234,235]
*P. oulorum*	Oral cavity (subgingival plaque)	[3]	Dentin caries Gingivitis Periodontitis Periodontal abscesses Alveolar osteitis (“dry socket”)	Rheumatoid arthritis* Systemic lupus erythematosus*	[122,124,138,141,157,163,206,217]
*P. pallens*	Oral cavity (gingival crevice, saliva)	[44]	Periodontitis Periodontal abscesses Halitosis Alveolar osteitis (“dry socket”) OSCC	Oropharyngeal and hypopharyngeal SCC Head and neck infections	[126,157,163,169,171,183,186,235]
*P. saccharolytica*	Supragingival plaque, periodontal pocket	[254]	Peri-implant mucositis	ND	[240]
*P. salivae*	Oral cavity (saliva; periodontitis)	[255]	Early childhood caries Endodontic infections Endodontic abscesses Periodontitis Periodontal abscesses OSCC	ND	[124,140,157,180,249]
*P. scopos*	Failing dental implant	[243]	Peri-implant mucositis	ND	[131]
*P. shahii*	Oral cavity	[255]	Peri-implant mucositis Halitosis	ND	[131,170,171]
*P. veroralis*	Oral cavity	[3]	Periodontitis Periodontal abscesses Peri-implant mucositis Halitosis Alveolar osteitis (“dry socket”)	ND	[124,128,131,157,163,171]
*Alloprevotella tannerae* (formerly *P. tannerae*)	Gingival crevice, periodontal pocket	[5]	Early childhood caries Dentin caries Endodontic infections Endodontic abscesses Periodontitis Severe odontogenic abscesses Peri-implantitis Halitosis OSCC	Oropharyngeal and hypopharyngeal SCC Laryngeal cancer Brain abscesses Pleural empyemas Early-onset rheumatoid arthritis*	[53,124,134,138–140,144,147,150,152–154,156,162,171,177,186,187,190–192,205,240]

ND = no data

OSCC = oral squamous cell carcinoma

### Periodontal and peri-implant diseases

In periodontitis-free pregnant women, mainly *P. nigrescens* was found to be associated with pregnancy-related gingivitis [36]. Interestingly, the estrogen level plays a role in the severity of inflammation at the gingival margin developed against plaque bacteria [120]. During pregnancy, the symbiotic composition is interrupted by the shifts towards anaerobes, including abundant *P. nigrescens, P. oris*, and *P. gingivalis* in plaque, and *Prevotella* oral taxon 313 and *P. melaninogenica* in saliva [121]. This transitional state of pregnancy-related dysbiosis usually returns to pre-gestational levels few months postpartum [120,121].

Chronic inflammation in gingival tissues can expose susceptible individuals to the initiation of periodontitis via alterations in subgingival bacterial communities. *P. oulorum* levels have been shown to be highly correlated with bleeding on probing scores during the transition from periodontal health to gingivitis [122]. *Prevotella* species can contribute to microbial dysbiosis and inflammation-regulated tissue destruction from its initiation to maturation. *P. denticola, P. multiformis*, and *P. oralis* have been connected to the severity of periodontal inflammation, correlating with increased age [123]. This kind of disrupted homeostasis may then result in the onset of disease as abundant *P. denticola, P. intermedia*, and *P. oralis* have been associated with deepening periodontal pockets [122,124]. Indeed, periodontitis is induced by bacterial infection, where *Prevotella* organisms as immunostimulatory oral bacteria can play a role as potential pathobionts or pathogens [10].

Proteomic and metagenomic studies have indicated a more complex microbial diversity in periodontitis patients and elevated abundances of *Prevotella* species; among salivary findings are *P. baroniae, P. enoeca, P. denticola, P. histicola, P. intermedia, P. melaninogenica, P. nigrescens, P. pallens, P. veroralis, A. tannerae*, and *A. rava*, but also *P. copri* and *P. stercorea*, rather known as inhabitants of the gut [125–128]. Questions may arise on how well the salivary microbiota represents the subgingival microbiota. However, the abundance of multiple subgingival plaque-related bacteria in saliva highly correlates with the progression of periodontitis [125].

Non-surgical periodontal therapy significantly decreased the levels of periodontitis-associated anaerobes, including *P. denticola, P. nigrescens*, and *A. tannerae*, in subgingival biofilms [129]. However, one should bear in mind that these bacteria do not fully disappear from the oral cavity; for example, *P. intermedia, P. melaninogenica, P. nigrescens*, and *P. veroralis* were detected in high abundances in saliva of periodontitis patients after 1 week, and *P. intermedia* and *P. veroralis* even after 8 weeks, of completed periodontal treatment [128]. Thus, high abundances of salivary *Prevotella* may contribute to the recurrence of periodontitis via bacterial recolonization of subgingival sites.

Although periodontal and peri-implant microbiomes share similarities in bacterial lineages, they represent microbiologically distinct ecosystems in health or disease [130,131]. In non-smokers, microbial abundances are reduced in peri-implant mucositis compared to peri-implant health, and a few health-associated species disappear along with a gain of various new species [131]. The species richness and consistency are similar between peri-implant mucositis and peri-implantitis. Several *Prevotella* taxa were identified among altered microbial communities. *P. scopos*, together with several unnamed taxa (HOT293, HOT299, HOT305 HOT306, and HOT315), were unique *Prevotella* findings during the shift from health to peri-implant mucositis. Then, *P. shahii, P. multisaccharivorax*, and unnamed taxa (HOT304 and HOT475) were recognized in peri-implant mucositis, *Prevotella* HOT526 during the shift from peri-implant mucositis to peri-implantitis, and *Prevotella* HOT515 and HOT820 in peri-implantitis [131]. Also, *P. intermedia, P. nigrescens*, and *P. denticola* have been reported at high numbers from peri-implantitis samples [132–134]. In peri-implantitis patients, deep peri-implant pockets, peri‐implant disease status, and partial edentulism were found to be associated with a high abundance of Gram‐negative anaerobic taxa, including members of the genus *Prevotella* [135]. The local environment affects the peri-implant biofilm composition; in comparison to shallow pockets, higher levels of *P. nigrescens* and *P. oris*, together with *F. nucleatum* and *Anaeroglobus geminatus*, were detected in deep pockets around dental implants. In 18 partially dentate and implant‐rehabilitated Chinese adults suffering from both periodontitis and peri-implantitis, the levels of *P. denticola, P. fusca*, and *P. multiformis* were markedly abundant in submucosal samples, whereas *P. dentalis* was more abundant in subgingival samples [136].

### Dental caries and root canal infections

High abundance of the genus *Prevotella* is associated with dental caries both in adults and children [137–140]. Intense and prolonged carbohydrate exposure alters the diversity of the microbial community by promoting the growth of surviving microorganisms under various acidic conditions, and the superficial parts are more acidic than the deepest sites of the caries lesion [138]. At a pH range of 4.5–5.0, lactobacilli act as anchor species, *Prevotella* taxa being the anchor species at a mid-range pH of 5.5–6.0. While *P. multisaccharivorax* and *P. histicola* were found in more acidic layers of dentinal caries, *P. oulorum* and *P. nigrescens* together with *A. tannerae* were prominent in mildly acidic and neutral pH regions of the caries lesion. Despite these species-specific variations regarding the pH gradient, approximately 60% of the dentinal caries-associated bacterial taxa, including eight *Prevotella* species, *P. baroniae, P. buccae, P. dentalis, P. intermedia, P. maculosa, P. marshii, P. oralis*, and *P. pleuritidis*, were non-discriminatory for pH changes, and thus, belonged to the substantial core microbiota [138]. This indicates that the presence of *Prevotella* in deep dentinal caries lesions reflects a suitable ecological niche rather than a contribution to caries progression [137].

According to a US study on dental caries experienced during early childhood, the diversity of caries-free sites and enamel caries lesions proved to be rather similar, whereas more taxa and enzyme activities were observed in dentin caries lesions, where *Scardovia wiggsiae* was most abundant, and also *P. denticola, P. oris*, and *P. oulorum* were among the most abundant species [141]. In an Irish study on the microbiome of severe early childhood caries (EEC) [140], higher relative abundance of *P. histicola* was detected both in deep dentinal caries lesions and in saliva from the same caries-active children, whereas *P. melaninogenica* and *P. salivae* dominated only in saliva. In a Chinese study, salivary levels of *P. denticola* and *Streptococcus mutans* were significantly higher in children with severe EEC than in caries-free children [142]. In supragingival plaque samples, collected from caries-active Kuwaiti children, the relative abundance of *P. melaninogenica* was significantly elevated [143].

Comparison between Romanian adolescents with a high prevalence of dental caries and Swedish adolescents with or without caries yielded a core microbiome of 24 species, including three *Prevotella* and both *Alloprevotella* species [144]. In Romanian adolescents with a high prevalence of caries and minimal dental care, the biofilm composition was dominated by ‘traditional’ caries pathogens, such as *S. mutans* and *Streptococcus sobrinus*, whereas their role was less pronounced in Swedish adolescents, who were under caries prevention programs. Interestingly, Swedish adolescents were typically characterized by an intake of sweets and having *P. maculosa* and *P. nigrescens* [144].

In supragingival plaque of elderly, the prevalence and relative abundance of *P. multisaccharivorax* was significantly increased and associated with root caries, suggesting its role as a true root caries pathogen [145,146]. Instead, *P. intermedia* was implicated as being probiotic, since it was highly prevalent and abundant at healthy, caries-free sites [146]. In a Japanese elderly population (n = 506), *P. melaninogenica, P. histicola*, and *P. pallens* were among 21 predominant OTUs in the tongue microbiota [42]. *P. histicola*, together with *Veillonella atypica, Streptococcus salivarius*, and *Streptococcus parasanguinis*, was more predominant in elderly with fewer teeth, a higher plaque index, and more dental caries experience.

Progressing caries lesions eventually reach the dental pulp, leading to pulpitis and pulpal necrosis. In Brazilian children, *P. intermedia* (96.9%), *P. nigrescens* (56.2%), and *P. denticola* (53.1%) were among the most prevalent species in primary teeth exhibiting pulp necrosis [147]. The microbiome of infectious root canals in their apical portions was primarily characterized as having *Prevotella* (17.9%) and *Bacteroidacea*e G-1 (14.3%), *P. oris* being the most numerous species [148]. Interestingly, elevated levels of *Prevotella* and *Porphyromonas* were found in symptomatic cases. In addition, *P. baroniae, P. intermedia, P. multisacchariavorax*, and *A. tannerae* predominate in apical periodontitis [149,150], and *P. buccae* and *P. intermedia* in radicular and residual cysts [151].

### Odontogenic infections

Odontogenic infections are typically of endodontic or periodontal origin. In acute apical abscesses, the most prevalent *Prevotella* taxa include *P. multisaccharivorax, P. intermedia, P. baroniae*, and *A. tannerae*, as well as occasional recoveries of *P. oralis* and *P. nigrescens* [152–156]. Significant variations in the microbiome can be, at least in part, explained by geographic/ethnic differences; for example, *P. nigrescens, A. tannerae*, and *F. nucleatum* were more prevalent in endodontic abscess samples from the USA than in those from Brazil [153]. Of the 138 clinical strains isolated from periodontal abscesses, the majority represented *Prevotella* taxa [157]. Of those, 1/3 were identified as *P. intermedia*, and around 1/10 as *P. nigrescens, P. melaninogenica, P. dentalis, P. denticola*, and *P. buccae* . Occasional recoveries included *P. fusca, P. histicola, P. multiformis, P. oris, P. oulorum, P. pallens, P. salivae, P. veroralis*, and *A. rava*. Recently, the microbiome compositions between abscess pus and the corresponding periodontal pocket (coronally from the abscess) and periodontally healthy gingival crevice samples were compared [158]. Based on the beta-diversity analyses, periodontal abscess pus and the periodontal pocket shared similar compositions. In comparison to the healthy gingival crevice, *P. intermedia* and *P. heparinolytica*, together with *P. gingivalis*, were the predominant findings and significantly more abundant in periodontal abscesses [158]. In 50 German patients hospitalized for severe odontogenic abscesses, the frequency of *Prevotella* was 17.7% in saliva (collected prior to the abscess incision) and 27.2% in the pus samples, being the most abundant genus in odontogenic abscesses [159]. In spreading odontogenic infections, members of the genus *Prevotella* are common, especially *P. buccae* and *P. oris* [160–162].

Alveolar osteitis, accompanied by poorly integrated blood clot in the alveolar socket, is a common complication after tooth extraction, resulting in severe postoperative pain. *Prevotella* has been found to be the most frequent genus in sockets with and without alveolar osteitis (22% and 18%, respectively) [163]. *Prevotella* recoveries from alveolar osteitis sites were only *P. nanceiensis, P. pleuritidis*, and *P. veroralis*, and occasionally, *P. copri, P. multiformis*, and *P. oulorum. P. melaninogenica* and *P. intermedia* were over-represented but *P. loescheii* and *P. salivae* were under-represented at alveolar osteitis sites compared to sockets without complications. In tooth sockets without complications, *P. bivia* and *P. marshii*, and occasional *P. aurantiaca, P. baroniae, P. bergensis*, and *P. oralis* were found [163].

### Diseases on oral mucosae

Oral lichen planus (OLP) is characterized as a chronic inflammatory condition affecting the oral mucosa with white striated, non-erosive or erosive lesions. In 13 OLP cases, the abundance of *P. melaninogenica* was significantly higher in comparison to samples from healthy mucosa [164]. Based on FISH analyses, epithelial and lamina propria invasion by *P. melaninogenica* was observed in OLP samples. As a novel aspect, *P. melaninogenica* was confirmed to activate the NF-kB signaling pathway after adhering to and invading macrophages, thus, indicating its role in the pathogenesis of OLP [164]. In recurrent aphthous stomatitis, *Prevotella* and *Alloprevotella* seem to be among the 10 most abundant genera, and the formation of aphthous ulcers may be due to the colonization of *Escherichia coli* and/or *Alloprevotella* [165]. Similarly, oral leukoplakia as a potentially malignant disorder was recently linked to salivary *Prevotella*, which was among the top 10 abundant taxa and among the most significant discriminating genera for leukoplakia [166].

### Sjögren’s syndrome

Sjögren’s syndrome (SS), an autoimmune disease, typically affects the salivary and lacrimal glands. In a Norwegian study, the composition of the salivary microbiome was examined in 45 women divided into primary-SS (p-SS), dry mouth (non-SS), and healthy control groups [167]. The mean relative abundance of the genus *Prevotella* was around 30%, and *P. histicola, P. melaninogenica*, and *P. salivae* were among the core microbiomes in all three groups. However, dysbiotic shifts in the salivary microbiota, including significantly reduced abundance of *P. pallens* and detection of *P. nanceiencis* and *P. intermedia*, were observed only in women with disturbed saliva secretion [167]. In a Korean study, comparing 25 women with p-SS and 25 controls with or without dry mouth, *P. melaninogenica* was highly associated (OR 22.4) with Sjögren’s syndrome [168]. Labial salivary gland biopsies with focal sialoadenitis revealed a high presence of bacteria, including *P. melaninogenica*, within the ductal cells and in the area of infiltration. In order to understand the bacterial involvement in the etiopathogenesis of Sjögren’s syndrome and in the functional and phenotypic changes in salivary glands, the authors used a human submandibular gland tumor cell model [168]. According to the results, *P. melaninogenica* increased and *P. histicola* decreased interferon (IFN)-λ production. Moreover, *P. melaninogenica* induced the deregulation of submandibular gland cells.

### Halitosis

Halitosis (oral malodor) is a common and multifactorial condition associated with volatile sulphur compounds (VSCs) of intra-oral or extra-oral origin. In preschool-aged children with halitosis, different *Prevotella* taxa have been reported; relative proportions of *P. pallens* and *A. rava* were dominant in tongue-coating samples [169], whereas the prevalence and relative abundance of salivary *P. shahii* were higher in halitosis children compared to children with normal VSC concentrations [170]. In Scottish adults with halitosis, *P. melaninogenica, P. pallens*, and *P. veroralis* were the predominant species on the tongue dorsum [171]. The comparison between the tongue microbiome of periodontally healthy Chinese individuals with and without intraoral halitosis revealed significantly higher relative percentages of *Prevotella* and *Alloprevotella* in halitosis samples [172]. Moreover, both genera correlated with increased VSC (here: H_2_S and CH_3_SH) values. This observation was in line with a Korean study where higher abundances of *Prevotella* and *Alloprevotella* in saliva correlated with amine metabolites, suggesting a causative mechanism via cadaverine and putrescine pathways [173].

## *Prevotella* in squamous cell carcinoma of the head and neck

The impact of the oral microbiota on cancer development has drawn remarkable attention among researchers. Dysbiotic bacterial communities are currently considered to play a role in carcinogenesis and cancer progression. Although the highest interest has been directed to *F. nucleatum* and *P. gingivalis* [174,175], there is considerable evidence available on the impact of various oral *Prevotella* species and *A. tannerae* on many cancer types in humans.

In the head and neck, squamous cell carcinoma (SCC), developing from the mucosal epithelium in the oral cavity, pharynx, and larynx, is among the most common cancer types globally [176]. Typical risk factors include an exposure to carcinogen-containing products (tobacco, alcohol, pollutants) as well as certain infectious agents (human papilloma and Epstein–Barr viruses). It has been shown that smokers have an altered bacterial composition on buccal mucosae, including *P. salivae, P. melaninogenica*, and *P. histicola* [34]. The authors suggested that smoking with increased nitrate intake favors nitrate-reducing species, which could be exposed to cancerous changes over the course of time. Also, poor oral hygiene, which favors the growth of moderate periodontal pathogens, e.g. *P. intermedia* and *A. tannerae*, may be linked to an increased risk for oral SCC [177]. Interestingly, *P. intermedia* was recognized at significantly elevated levels from tumor sites located in the gingiva but also in the specific mucosa of the tongue, or buccal and mouth floor mucosae [178].

Recently, Gopinath and co-workers [179] studied the oral bacteriome of oral SCC patients, collecting three types of samples from the oral cavity: ‘whole mouth fluid’ (including components of saliva, mucosal transudate, inflammatory exudate, and gingival crevicular fluid), a swab of the cancer surface, and cancer tissue from the body of the tumor. First, it was shown that the oral bacteriome, based on the whole mouth fluid sample, and its metabolic activity in cancer patients are clearly distinct from those of healthy controls. Further, the authors found that the bacteriome differed significantly between the surface and deeper part of the tumor. *Prevotella* organisms were located in deep cancerous tissue, and their increase in abundance, being in line with previous reports, could indicate a role in cancer progression [179]. Studies presenting *Prevotella* species-level data have found *P. intermedia, P. loescheii*, and *P. salivae* enriched in cancer tissues [178,180,181]. Altered salivary microbiomes with increased abundance of *P. multisaccharivorax* [182], *P. melaninogenica, P. nanceiensis, P. nigrescens*, and *P. pallens* [183], and *P. intermedia, P. oris*, and *A. tannerae* [177] have been reported from oral SCC patients. In the latter study, however, *P. melaninogenica* and *P. histicola* were slightly more abundant and *P. pallens* equally abundant in controls [177]. Interestingly, reduced proportions of mucosa-associated *P. melaninogenica* and *P. histicola* were observed in the salivary microbiota after surgical tongue resection compared to pre-surgery salivary samples of tongue cancer patients [184].

In tonsillar crypts, the genus *Prevotella*, in addition to *Veillonella* and *Fusobacterium*, showed the highest abundance in patients with tonsillar SCC, differing from tonsillar findings in obstructive sleep apnea patients used as controls [185]. These bacterial alterations were interpreted as being connected to conditions induced by tumor growth. At the species level, *P. melaninogenica* was among the top-10 tumor-predictive species, and this was seen to be consistent with its increased presence in saliva, as reported in previous studies [185]. In contrast, an Indian study, investigating alterations in the salivary microbiota of oropharyngeal and hypopharyngeal SCC patients, reported reduced abundances of *P. melaninogenica, P. nanceiensis, P. nigrescens, P. pallens*, and *A. tannerae* both in oropharyngeal and hypopharyngeal cancer patients [186]. Instead, the study reported, for the first time, the presence and increased abundance of *P. copri*, an intestinal *Prevotella* species, in these cancer types.

In the throat, the three most frequent genera with proportions higher than 0.01%, in descending order, were *Streptococcus, Fusobacterium*, and *Prevotella* [187]. Bacterial community compositions and abundances differed between tissue samples (two collection sites: upper and lower epiglottis) from laryngeal cancer patients and those from controls with vocal cord polyps. Concerning *Prevotella*, increased prevalences of *P. intermedia, P. nigrescens, P. oris*, and *A. tannerae* were found in cancer patients [187]. Another Chinese study investigated bacterial communities on mucous membranes of the vocal cords [188], targeting to reveal differences between glottic laryngeal SCC and adjacent non-tumor tissues and vocal cord polyp tissues (controls). The genera *Fusobacterium, Alloprevotella*, and *Prevotella* proved to be enriched in tumor tissue.

A follow-up study looked for changes in microbial community profiles of the larynx prior to laryngectomy and 1 week and 24 weeks thereafter [189]. Smoking and alcohol consumption strongly affected the community structure; a positive association was found between the drinking index and abundance of *Prevotella* in cancer patients prior to laryngectomy. The abundance of *Alloprevotella* was significantly reduced at 24 weeks post-surgery. It has been speculated whether alcohol consumption influences the microbial community structure of the throat, which could then lead to laryngeal carcinogenesis.

## Non-oral infections with emphasis on oral *Prevotella*

### Abscesses and soft tissue infections

Oral bacteria are commonly reported from various types of abscesses at different body sites, including the brain and lungs. A part of brain abscesses was polymicrobial, including microaerophilic and anaerobic species, especially *Aggregatibacter aphrophilus, Streptococcus intermedius*, and *F. nucleatum* [190,191]. The role of *F. nucleatum* as one of the primary pathogens in brain abscesses with strictly anaerobic species was suggested to be due to its key role in coaggregation and biofilm formation as seen in oral infections [191]. Hematogenous spread of oral pathogens and dysbiotic biofilms through the skull bone may be an important infectious route for their access into the venous system of the brain. Thus, bacterial brain abscesses could be of oral or sinus origin. These comprehensive studies on the microbiology of brain abscesses, using advanced molecular methods, recognized various *Prevotella* species in pus specimens as follows: *P. baroniae, P. denticola, P. heparinolytica P. intermedia, P. nigrescens, P. oris, P. pleuritidis*, and *A. tannerae* as well as not-yet-named *Prevotella* HOT 314 and HOT 317 [190,191].

Polymicrobial findings, clustering *F. nucleatum* with other anaerobic species typical in oral biofilms, encouraged a Norwegian research group to take a thorough look at bacterial recoveries from pleural empyemas, due to similarities between the brain and lungs as being highly oxygenated organs, and to compare the bacterial compositions of these purulent infections [192]. Pleural fluid samples were examined using massive parallel sequencing of partial *16S rRNA* and *rpoB* genes, i.e. the same method as used for brain abscesses [191]. Again, *F. nucleatum* and *S. intermedius* were dominant findings in empyema specimens similarly to previous brain specimens. By analyzing 27 empyemas with poorly described etiology and 25 brain abscesses where oral or sinus origin was assumed, several anaerobic species, among those *P. oris, P. pleuritidis, Prevotella* HMT 317, and *A. tannerae*, were recovered from both brain abscesses and pleural empyemas, while *P. buccae* findings were only from the latter one [192].

In the oropharynx during recurrent tonsillitis, a shift in bacterial composition has occurred, resulting in an increased abundance of *P. melaninogenica/P. histicola* [29]. Oral members of the genus *Prevotella* are among the polymicrobial consortia of peritonsillar abscesses with or without complications [193,194]. In a study on abscesses of the head and neck region, 33 clinical *Prevotella* isolates were identified as *P. melaninogenica, P. oralis, P. buccae, P. intermedia*, and *P. denticola* with a biochemical test kit [195]. In breast abscesses, *P. buccae, P. bivia*, and *P. bergensis* have been among anaerobic bacteria [196]. Interestingly, a metronidazole-resistant *P. buccae* strain was reported from an infected breast cyst [197]. Also, the genus *Prevotella* seems to play a role in hidradenitis suppurativa (acne inversa) [198,199]. This is a chronic inflammatory skin disease, where purulent lesions are formed in sweltering regions, including the underarms and under the breasts. Anaerobes predominated in 149 samples from lesions compared to 175 samples from unaffected skinfolds, and especially Gram-negative, strictly anaerobic bacteria were enriched; *Prevotella* and *Porphyromonas* were detected nearly ubiquitously and were the most abundant bacterial genera in lesions where *P. intermedia, P. nigrescens*, and *P. melaninogenica* were named [198]. In osteomyelitic bone specimens collected from 17 diabetic foot infections, *Prevotella* proved to be the most abundant genus [200]. *P. denticola, P. fusca*, and *P. intermedia*, typical *Prevotella* species of oral origin, and *P. jejuni*, were detected, and all of them positively correlated with the duration of the foot infection.

Some novel *Prevotella* species without knowledge of their preferred habitat have been among recoveries, especially from pus specimens. For example, the description of *P. pleuritidis* was performed based on a single strain from pleural fluid [201], and since then, its presence was reported also from lung and liver abscesses [202,203]. Noteworthy are the frequently abundant findings of *P. pleuritidis* among the oral microbiota of the study populations from the United Arab Emirates and Sweden: in subgingival biofilms of heavy smokers [204] and in saliva of early-onset rheumatoid arthritis patients [205], respectively. In addition, increased proportions of *P. pleuritidis* subgingivally have been reported from systemic lupus erythematosus patients with periodontitis [206] and from post-extraction sockets with alveolar osteitis [163]. These results strongly indicate that *P. pleuritidis* is an oral species. The strains of three individuals characterized for the description of *P. nanceiensis* originated from the pus of a lung abscess, broncho-alveolar fluid, and blood [207]. Since then, *P. nanceiensis* has been recovered from the adenoid specimens of children with middle ear effusion [208], health-associated tongue coating microbiome of children [170], as well as from saliva of children with a gluten-free diet due to celiac disease [209], and from saliva [128] and alveolar osteitis [163] of adults, indicating *P. nanceiensis* as being of oral origin. In the description of two novel *Prevotella* species, the *P. vespertina* strain originated from an unspecified abscess located at the upper respiratory tract [210] and the *P. illustrans* strain from an oropharyngeal abscess [211]. The eight strains described as *P. bergensis* were isolated from infections of the skin and soft-tissue abscesses [212], and recently, it was found in a breast abscess [196]. The original description of *P. timonensis* was based on a single strain from a breast abscess [213]. Among 84 clinical *P. timonensis* strains collected in a university hospital during a 10-year period, most strains came from cutaneous/soft tissue and bone infections, but also from genitourinary tract specimens [214]. Despite being originally isolated from a breast abscess, it was assumed that the habitat of *P. timonensis* is below the waistline. In contrast, *P. jejuni*, originally isolated from the jejunum of a celiac child, seems to be among the resident members of the oral cavity [33,37,38].

## Potential involvement of oral *Prevotella* in chronic pathological conditions outside the mouth

### Rheumatic diseases

To date, *P. gingivalis* is widely considered a major oral species associated with rheumatoid arthritis (RA), a common autoimmune disease. However, by comparing subgingival microbiota and its abundance and diversity between new-onset RA, chronic RA, and healthy individuals, it was shown that the overall exposure to *P. gingivalis* did not differ between the RA and control groups, whereas *Prevotella* (and *Leptotrichia*) species were characteristic for separating the newly-onset RA group regardless of periodontal status [215]. Based on these results, Scher and co-workers [215] appealed for further studies on these anaerobic taxa. Since then, the relative abundance of *Prevotella* has been shown to be increased in saliva of patients with early RA but also at-risk individuals, and an interesting observation was a low abundance for *P. gingivalis* also here [216]. Indeed, there is now increasing evidence available on specific *Prevotella* organisms in this destructive inflammatory disease of synovial joints. Significant differences were observed in subgingival bacterial communities in RA patients who had high bacterial loads and diverse microbiotas, even in periodontitis-free individuals, in comparison to controls [217]. Among elevated *Prevotella* findings were *P. melaninogenica, P. denticola, P. histicola, P. nigrescens, P. oulorum*, and *P. maculosa*. Concerning *P. melaninogenica*, the finding is in agreement with its increased abundance in rheumatoid and osteoarthritis individuals detected by Chen et al. [218], but in contrast to that reported by Lehenaff et al. [219]. Recently, a Swedish study group [205] focused on species-level differences of the oral microbiota between 61 untreated early-onset RA patients and their healthy age- and sex-matched controls, also taking their periodontal status into account. *P. pleuritidis, P. dentalis, P. intermedia*, two *Prevotella* clones (HMT 317 and HMT 300), and *A. tannerae* were among the top 20 most influential taxa for separating the cases and controls. Influential *Prevotella* species in saliva of early-onset RA individuals without deepened periodontal pockets were *P. denticola* and *P. nigrescens*, and in those with deepened pockets (pocket depth >6 mm) were *P. dentalis, P. intermedia*, and *P. melaninogenica* [205]. Although *P. pleuritidis* was more abundant in early-onset RA patients, it was not associated with periodontal status.

The link between systemic lupus erythematosus (SLE), which is an autoimmune disease affecting joints, and periodontitis was studied in 52 SLE patients and their age- and sex-matched controls by Correa and co-workers [206]. It is assumed that this systemic disease alters the composition of subgingival biofilms, exposing them to the development of periodontitis via dysbiotic microbiota and deterioration of SLE. Indeed, SLE patients had a reduced microbial diversity and higher pathogen load, among those *P. nigrescens, P. oris*, and *P. oulorum*, regardless of the periodontal status. The relative abundances of *P. nigrescens* correlated with higher SLE index scores (SLE damage and activity), *P. oris* with SLE duration, *P. melaninogenica* with increased C-reactive protein levels, *P. denticola* with elevated neutrophils, and *P. oulorum* with elevated lymphocytes [206].

### Neurological diseases

The impact of oral health on neurodegenerative diseases has emerged along with research interest in the potential inflammatory etiology of Alzheimer's disease (AD), and some evidence exists on *Prevotella* organisms in this context. Using a longitudinal study design, serum IgG levels to seven periodontal pathogens, including *P. intermedia*, were analyzed in 158 study participants who were cognitively intact at baseline and were then followed for around 10 years [220]. Of the 81 individuals experiencing either AD or mild cognitive impairment, significantly increased IgG levels to *P. intermedia* and *F. nucleatum* in particular were found prior to the diagnosis of AD. A recent study using serum antibody data from the NHANES study population showed that AD’s mortality risk was increased in individuals with high IgG levels for several periodontal pathogens, including *P. intermedia, P. nigrescens*, and *P. melaninogenica* [221]. The link to periodontitis-associated bacteria was stronger for older adults.

In pioneer studies from the beginning of the 21st century, intestinal microorganisms, especially anaerobic bacterial taxa, were suggested to play a potential role in autism, a neurodevelopmental disorder [222,223]. Interestingly, while non-clostridial anaerobic and microaerophilic bacteria were common in aspirates collected from the small intestine of autistic children, they were absent from their controls [222]. At the phylum level, important differences between the autistic and neurotypical children have been observed especially within *Firmicutes, Bacteroidetes, Actinobacteria*, and *Proteobacteria* [223]. Now, a few studies have looked for the role of the oral microbiota in this context, and only very limited data are available on *Prevotella* organisms. Qiao and co-workers [224] analyzed bacterial taxa at the genus level in dental plaque and saliva of children with and without autism spectrum disorder (ASD). Microbial alterations were observed, including decreased rates of the genera *Prevotella* and *Alloprevotella* in saliva, but also a reduction of the family *Prevotellaceae* co-occurrence network in dental plaque of ASD children [224]. Recently, the tongue microbiome was examined to assess whether there could be a link between the bacterial composition on the tongue surface and ASD [225]. The genus *Prevotella* and the species *P. melaninogenica* were among the most abundant findings in studied children; however, no significant differences were detected between ASD and control children. The microbiology of these neurodevelopmental disorders is still at an early phase to be assessed.

The potential role of bacterial communities of the oral cavity and oropharynx in individuals with psychiatric disorders is of current research interest. In salivary samples from young adults with and without depression, the most prevalent were *Neisseria* spp. and *P. nigrescens*, but distinct bacterial abundance patterns were found between the cases and controls [226]. Smoking was recognized as a contributing factor for alterations in the salivary microbiome. While *P. nigrescens* was more abundant in depressive individuals, several other known species like *P. nanceiensis, P. oris*, and *A. rava* were reduced and more abundant in controls [226]. A similar reduced abundance of the genus *Prevotella* in the oropharyngeal microbiome was reported for individuals with schizophrenia and mania as well as with major depressive disorder in comparison to non-psychiatric controls [227]. The altered beta-diversity in individuals with schizophrenia and mania was independent of smoking. No significant associations were observed between the levels of identified bacterial taxa and psychiatric symptoms.

Some fragmentary information is available on the involvement of *Prevotella* organisms in vascular conditions in the intracranial region. Regarding ischemic stroke, the genus *Prevotella* was found to be enriched in the patient group with poor treatment outcome [228]. DNA of *P. intermedia* has been recovered from ruptured intracranial aneurysm walls [229] and in culture of cerebrospinal fluid from a patient of intracranial mycotic aneurysm [230]. A metronidazole-resistant *P. loescheii* strain has been isolated from subdural hematoma [231].

## Summary

Research into the genus *Prevotella* has increased considerably over the past years. *Prevotella* has a wide intra-genus variation, which leads to a variety of properties between *Prevotella* species. Interest is focused on their involvement as commensals and potentially pathogenic organisms at different locations of the human body. In the oral cavity, *Prevotella* are important members of bacterial communities on various mucosal surfaces and in saliva as well as in dental biofilms above and below the gumline. However, knowledge of individual *Prevotella* species other than *P. intermedia* and *P. nigrescens* and their role in keeping homeostasis or interfering in dysbiotic biofilms is still limited. As regards *P. melaninogenica*, on the one hand, it belongs to the core oral bacteriome in individuals with healthy oral tissues but, on the other hand, it seems to be involved in many pathogenic conditions inside and outside the mouth. Is it a bystander or an active player in these cases? Also, the influence of behavioral and lifestyle factors on oral *Prevotella* is not clear. For example, an interesting question whether diet could have an impact on oral *Prevotella* species remains to be answered. Furthermore, increasingly attractive is the potential link of oral *Prevotella* organisms to systemic conditions not usually connected to microbes.
